# 2-Amino-4,6-dimeth­oxy­pyrimidin-1-ium *p*-toluene­sulfonate

**DOI:** 10.1107/S160053681103755X

**Published:** 2011-09-17

**Authors:** Sundaramoorthy Gomathi, Packianathan Thomas Muthiah

**Affiliations:** a School of Chemistry, Bharathidasan University, Tiruchirappalli 620 024, Tamilnadu, India

## Abstract

In the title salt, C_6_H_10_N_3_O_2_^+^·C_7_H_7_O_3_S^−^, the 2-amino-4,6-dimeth­oxy­pyrimidinium cation inter­acts with the sulfonate group of the *p*-toluene­sulfonate anion *via* a pair of N—H⋯O hydrogen bonds, forming a cyclic hydrogen-bonded *R*_2_^2^(8) motif, which in the crystal is linked by further intemolecular N—H⋯O hydrogen bonds, forming supra­molecular chains along the *c* axis. Furthermore, neighboring chains are inter­linked *via* weak C—H⋯O hydrogen bonds and C—H⋯π inter­actions, forming layers.

## Related literature

For background to crystal engineering and supra­molecular chemistry, see: Desiraju (1989 ▶). For the role of amino­pyrimidine–carboxyl­ate inter­actions in protein-nuleic acid recognition and protein-drug binding, see: Hunt *et al.* (1980 ▶); Baker & Santi (1965 ▶). For the role of sulfate–protein inter­actions, see: Pflugrath & Quiocho (1985 ▶); Jacobson & Quiocho (1988 ▶). For information on carb­oxy­lic acid inter­actions with a 2-amino heterocyclic ring system, see: Etter & Adsmond (1990 ▶); Lynch & Jones (2004 ▶); Allen *et al.* (1998 ▶). For a survey of hydrogen-bonding patterns involving sulfonate salts, see: Haynes *et al.* (2004 ▶). For hydrogen-bonding patterns involving sulfonate groups in biological systems and metal complexes, see: Russell *et al.* (1994 ▶); Cai *et al.* (2001 ▶). For hydrogen-bond motifs, see: Bernstein *et al.* (1995 ▶); Etter (1990 ▶). For related structures, see: Low *et al.* (2002 ▶); Arora & Sundaralingam (1971 ▶); Balasubramani *et al.* (2007 ▶); Hemamalini *et al.* (2005 ▶); Thanigaimani *et al.* (2007 ▶, 2008 ▶); Ebenezer & Muthiah (2010 ▶).
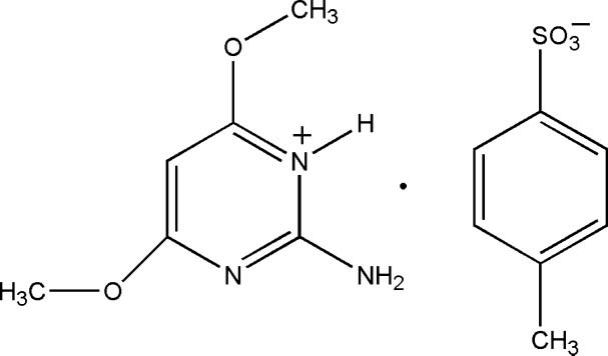


## Experimental

### 

#### Crystal data

C_6_H_10_N_3_O_2_^+^·C_7_H_7_O_3_S^−^
*M* *_r_* = 327.37
Orthorhombic, 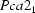

*a* = 15.2116 (2) Å
*b* = 12.1422 (2) Å
*c* = 8.3497 (1) Å
*V* = 1542.21 (4) Å^3^
*Z* = 4
Mo *K*α radiation
μ = 0.24 mm^−1^
*T* = 296 K
0.20 × 0.18 × 0.15 mm

#### Data collection

Bruker SMART APEXII CCD area-detector diffractometer
Absorption correction: multi-scan (*SADABS*; Bruker, 2008 ▶) *T*_min_ = 0.954, *T*_max_ = 0.965
35029 measured reflections
5264 independent reflections
4257 reflections with *I* > 2σ(*I*)
*R*_int_ = 0.032

#### Refinement

*R*[*F* ^2^ > 2σ(*F* ^2^)] = 0.035
*wR*(*F* ^2^) = 0.098
*S* = 1.04
5264 reflections
202 parameters
1 restraint
H-atom parameters constrained
Δρ_max_ = 0.21 e Å^−3^
Δρ_min_ = −0.23 e Å^−3^
Absolute structure: Flack (1983 ▶) 2449, Friedel pairs
Flack parameter: −0.01 (6)

### 

Data collection: *APEX2* (Bruker, 2008 ▶); cell refinement: *SAINT* (Bruker, 2008 ▶); data reduction: *SAINT*; program(s) used to solve structure: *SHELXS97* (Sheldrick, 2008 ▶); program(s) used to refine structure: *SHELXL97* (Sheldrick, 2008 ▶); molecular graphics: *PLATON* (Spek, 2009 ▶) and *POV-RAY* (Cason, 2004) ▶; software used to prepare material for publication: *PLATON*.

## Supplementary Material

Crystal structure: contains datablock(s) global, I. DOI: 10.1107/S160053681103755X/lh5333sup1.cif

Structure factors: contains datablock(s) I. DOI: 10.1107/S160053681103755X/lh5333Isup2.hkl

Supplementary material file. DOI: 10.1107/S160053681103755X/lh5333Isup3.cml

Additional supplementary materials:  crystallographic information; 3D view; checkCIF report

## Figures and Tables

**Table 1:** Hydrogen-bond geometry (Å, °) *Cg* is the centroid of the C9–C14 ring.

*D*—H⋯*A*	*D*—H	H⋯*A*	*D*⋯*A*	*D*—H⋯*A*
N1—H1⋯O4	0.86	1.90	2.7441 (16)	169
N2—H2*A*⋯O3^i^	0.86	2.20	3.0233 (19)	161
N2—H2*B*⋯O3	0.86	2.12	2.9398 (18)	159
C8—H8*B*⋯O5^ii^	0.96	2.37	3.248 (2)	152
C7—H7*A*⋯*Cg*^iii^	0.96	2.96	3.7815 (18)	145

Symmetry codes: (i) 
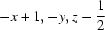
; (ii) 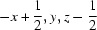; (iii) 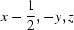.

**Fig. 1.**
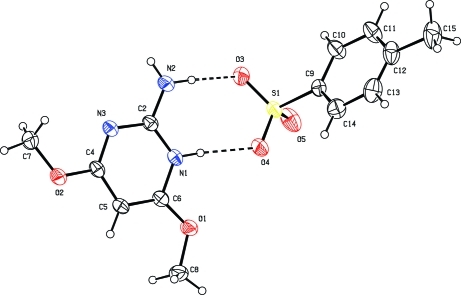
The asymmetric unit of (I), showing 30% probability displacement ellipsoids. Dashed lines indicate hydrogen bonds.

**Fig. 2.**
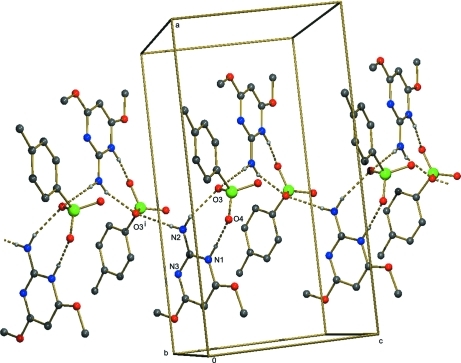
A view of supramolecular chain running along the c axis. [symmetry code: (i)1 - x,-y,-1/2 + z]

## supplementary crystallographic information

### Comment

A study of non-covalent interactions, such as hydrogen bonding, plays a key role in molecular recognition and crystal engineering (Desiraju, 1989). Pyrimidines and aminopyrimidine derivatives are biologically important compounds and they manifest themselves in nature as components of nucleic acids. Some aminopyrimidine derivatives are used as antifolate drugs (Hunt *et al.*, 1980; Baker & Santi, 1965). Their interactions with carboxylic acids are of utmost importance since they are involved in protein-nucleic acid recognition and drug-protein recognition processes, where the pyrimidine moiety of a drug forms hydrogen bonding with the carboxyl group of the protein. Aminopyrimidines readily pair up with carboxylic acids to form a wide variety of 1:1 adducts with mono and dicarboxylic acids (Etter & Adsmond, 1990). The *R*_2_^2^(8) motif is a robust synthon which is frequently observed when a carboxylic acid interacts with a 2-amino heterocyclic ring system (Lynch & Jones, 2004). This motif is also recognized to be one of the top 5 motifs among the 24 commonly occurring motifs in crystal structures (Allen *et al.*, 1998). In a sulfate-binding protein, the sulfate anion is bound mainly by seven hydrogen bonds, five of which are from the main chain peptide NH groups (Pflugrath & Quiocho, 1985; Jacobson & Quiocho, 1988). Hydrogen bonding patterns involving sulfonate groups in biological systems and metal complexes are of current interest (Russell *et al.*, 1994; Cai *et al.*, 2001). Such interactions can be used for designing supramolecular architectures.

The crystal structures of 2-amino-4, 6-dimethoxy pyrimidine (Low *et al.*, 2002) and *p*-toluene sulfonic acid monohydrate (Arora & Sundaralingam, 1971) have already been reported. Investigations of a fairly large number of crystal structure of 2-amino-4,6-dimethoxy/dimethyl pyrimidine salts and co crystals involving carboxylates (Thanigaimani *et al.*, 2007; Thanigaimani *et al.*, 2008; Ebenezer & Muthiah, 2010) and a few sulfonates (Balasubramani *et al.*, 2007; Hemamalini *et al.*, 2005) have already been reported from our laboratory. They reveal the formation of certain robust motifs and a variety of supramolecular architectures. A survey by Haynes *et al.* (2004) on the sulfonate salts, revealed various hydrogen bonding patterns and their preferences with specific functional groups. As part of our investigation to gain more insight into hydrogen bonding interactions involving aminopyrimidine and sulfonates, the crystal structure of title compound is presented herein.

The asymmetric unit of the title compound (I) (Fig. 1) contains one 2-amino-4,6-dimethoxypyrimidinium cation and one *p*-toluenesulfonate anion. The 2- amino-4,6-dimethoxy pyrimidinium cation is protonated at N1. Protonation of the pyrimidine base on the N1 site is reflected by an increase in bond angle. The C2—N3—C4 angle of the unprotonated atom N3 is 116.52 (12)° while for protonated atom N1, the C2—N1—C6 angle is 120.64 (11)°. The sulfonate group of the *p*-toluenesulfonate anion interacts with 2-amino-4,6-dimethoxypyrimidinium cation *via* a pair of N—H···O hydrogen bonds, forming a hydrogen bonded ring motif with graph-set notation *R*_2_^2^(8) (Etter, 1990; Bernstein *et al.*, 1995). The sulfonate group mimics the carboxylate anion's mode of association, which is more commonly seen when binding with 2-aminopyrimidines. The *R*_2_^2^(8) motif links O3 and O4 atoms of sulfonate anion with the protonated atom N1 and the 2- amino group of the pyrimidinium cation.

This motif is further interlinked by an N—H···O hydrogen bond, involving 2- amino group of the 2-amino-4,6-dimethoxy pyrimidinium cation and O3^i^ (symmetry code: i - *x*,-*y*,-1/2 + *z*)) atom of *p*-toluenesulfonate anion to form a supramolecular chain along the *c* axis (Fig. 2). The neighboring supramolecular chain is further interlinked *via* C—H···O hydrogen bond involving a methoxy group (C8) of cation and O5^ii^ (symmetry code: 1/2 - *x*, *y*, -1/2 + *z*) atom of sulfonate anion.  Thus intermolecular hydrogen bonds generate a 2-D supramolecular network. The crystal structure is further stabilized by C—H··· π interaction. The C—H···π interaction is observed between the methoxy group (C7—H7A) of pyrimidinium cation with phenyl ring of *p*-toluenesulfonate anion (C—H···π = 3.7815 (18) Å, 145°). The identification of such supramolecular patterns will help us design and construct preferred hydrogen bonding patterns on drug like molecules.

### Experimental

A hot ethanolic solution (20 ml) of 2-amino-4,6-dimethoxypyrimidine (38 mg, Aldrich) and *p*-toluene sulfonic acid (47 mg, Loba Chemie) was warmed for half an hour over a water bath. The mixture was cooled slowly and kept at room temperature; after a few days, colorless prismatic crystals were obtained.

### Refinement

All hydrogen atoms were positioned geometrically and were refined using a riding model. The N—H and C—H bond lengths are 0.86 and 0.93–0.96 Å, respectively [*U*_iso_(H)=1.2-1.5*U*_eq_ (parent atom)].

### Crystal data

**Table:**

C_6_H_10_N_3_O_2_^+^·C_7_H_7_O_3_S^−^	*F*(000) = 688
*M**_r_* = 327.37	*D*_x_ = 1.410 Mg m^−^^3^
Orthorhombic, *P**c**a*2_1_	Mo *K*α radiation, λ = 0.71073 Å
Hall symbol: P 2c -2ac	Cell parameters from 5264 reflections
*a* = 15.2116 (2) Å	θ = 1.7–31.9°
*b* = 12.1422 (2) Å	µ = 0.24 mm^−^^1^
*c* = 8.3497 (1) Å	*T* = 296 K
*V* = 1542.21 (4) Å^3^	Prism, colourless
*Z* = 4	0.20 × 0.18 × 0.15 mm

### Data collection

**Table:**

Bruker SMART APEXII CCD area-detector diffractometer	5264 independent reflections
Radiation source: fine-focus sealed tube	4257 reflections with *I* > 2σ(*I*)
graphite	*R*_int_ = 0.032
φ and ω scans	θ_max_ = 31.9°, θ_min_ = 1.7°
Absorption correction: multi-scan (*SADABS*; Bruker, 2008)	*h* = −21→22
*T*_min_ = 0.954, *T*_max_ = 0.965	*k* = −17→18
35029 measured reflections	*l* = −12→12

### Refinement

**Table:**

Refinement on *F*^2^	Secondary atom site location: difference Fourier map
Least-squares matrix: full	Hydrogen site location: inferred from neighbouring sites
*R*[*F*^2^ > 2σ(*F*^2^)] = 0.035	H-atom parameters constrained
*wR*(*F*^2^) = 0.098	*w* = 1/[σ^2^(*F*_o_^2^) + (0.0621*P*)^2^ + 0.0019*P*] where *P* = (*F*_o_^2^ + 2*F*_c_^2^)/3
*S* = 1.04	(Δ/σ)_max_ = 0.001
5264 reflections	Δρ_max_ = 0.21 e Å^−^^3^
202 parameters	Δρ_min_ = −0.23 e Å^−^^3^
1 restraint	Absolute structure: Flack (1983) 2449, Friedel pairs
Primary atom site location: structure-invariant direct methods	Flack parameter: −0.01 (6)

### Special details

**Table:**

Geometry. Bond distances, angles *etc*. have been calculated using the rounded fractional coordinates. All su's are estimated from the variances of the (full) variance-covariance matrix. The cell e.s.d.'s are taken into account in the estimation of distances, angles and torsion angles
Refinement. Refinement on *F*^2^ for ALL reflections except those flagged by the user for potential systematic errors. Weighted *R*-factors *wR* and all goodnesses of fit *S* are based on *F*^2^, conventional *R*-factors *R* are based on *F*, with *F* set to zero for negative *F*^2^. The observed criterion of *F*^2^ > σ(*F*^2^) is used only for calculating -*R*-factor-obs *etc*. and is not relevant to the choice of reflections for refinement. *R*-factors based on *F*^2^ are statistically about twice as large as those based on *F*, and *R*-factors based on ALL data will be even larger.

### Fractional atomic coordinates and isotropic or equivalent isotropic displacement parameters (Å^2^)

**Table:**

	*x*	*y*	*z*	*U*_iso_*/*U*_eq_	
O1	0.19507 (6)	0.22303 (9)	0.38465 (14)	0.0511 (3)	
O2	0.11421 (6)	−0.06832 (9)	0.04130 (13)	0.0501 (3)	
N1	0.29344 (7)	0.11712 (9)	0.26107 (13)	0.0402 (3)	
N2	0.40346 (7)	0.01635 (11)	0.14147 (18)	0.0496 (4)	
N3	0.25982 (7)	−0.03173 (10)	0.08987 (14)	0.0404 (3)	
C2	0.31858 (9)	0.03320 (10)	0.16420 (17)	0.0390 (3)	
C4	0.17567 (9)	−0.00833 (12)	0.11384 (17)	0.0403 (4)	
C5	0.14437 (8)	0.07733 (12)	0.21022 (17)	0.0428 (3)	
C6	0.20693 (8)	0.13947 (11)	0.28454 (15)	0.0400 (4)	
C7	0.14081 (10)	−0.16405 (13)	−0.0479 (2)	0.0540 (4)	
C8	0.10582 (10)	0.25880 (15)	0.4142 (2)	0.0563 (5)	
S1	0.50030 (2)	0.23323 (3)	0.42863 (6)	0.0428 (1)	
O3	0.52077 (8)	0.12267 (9)	0.37468 (16)	0.0570 (4)	
O4	0.41176 (7)	0.26565 (10)	0.3831 (2)	0.0647 (5)	
O5	0.51823 (10)	0.24747 (13)	0.59809 (18)	0.0739 (5)	
C9	0.57224 (9)	0.32344 (11)	0.32795 (17)	0.0422 (3)	
C10	0.66162 (10)	0.30482 (15)	0.3425 (2)	0.0591 (5)	
C11	0.72049 (13)	0.37635 (17)	0.2707 (3)	0.0705 (6)	
C12	0.69171 (15)	0.46724 (14)	0.1854 (2)	0.0648 (6)	
C13	0.60373 (15)	0.48291 (15)	0.1698 (3)	0.0698 (6)	
C14	0.54237 (12)	0.41230 (13)	0.2414 (2)	0.0601 (5)	
C15	0.7578 (2)	0.54823 (18)	0.1173 (3)	0.0919 (9)	
H1	0.33270	0.15660	0.30810	0.0480*	
H2A	0.42080	−0.03590	0.07950	0.0600*	
H2B	0.44140	0.05760	0.18870	0.0600*	
H5	0.08460	0.09110	0.22280	0.0510*	
H7A	0.16740	−0.21650	0.02330	0.0810*	
H7B	0.18250	−0.14290	−0.12860	0.0810*	
H7C	0.09030	−0.19660	−0.09800	0.0810*	
H8A	0.07310	0.19980	0.46210	0.0840*	
H8B	0.07880	0.27950	0.31480	0.0840*	
H8C	0.10640	0.32090	0.48540	0.0840*	
H10	0.68200	0.24450	0.40020	0.0710*	
H11	0.78050	0.36310	0.27980	0.0850*	
H13	0.58360	0.54230	0.10970	0.0840*	
H14	0.48240	0.42530	0.23060	0.0720*	
H15A	0.72930	0.59500	0.04060	0.1380*	
H15B	0.80450	0.50860	0.06570	0.1380*	
H15C	0.78160	0.59230	0.20230	0.1380*	

### Atomic displacement parameters (Å^2^)

**Table:**

	*U*^11^	*U*^22^	*U*^33^	*U*^12^	*U*^13^	*U*^23^
O1	0.0369 (5)	0.0605 (6)	0.0559 (6)	0.0002 (4)	−0.0028 (4)	−0.0136 (5)
O2	0.0340 (4)	0.0573 (6)	0.0589 (6)	−0.0034 (4)	−0.0075 (4)	−0.0086 (5)
N1	0.0323 (5)	0.0477 (6)	0.0407 (5)	−0.0051 (4)	−0.0035 (4)	0.0014 (4)
N2	0.0311 (5)	0.0558 (7)	0.0619 (8)	−0.0011 (5)	−0.0030 (5)	−0.0073 (6)
N3	0.0325 (5)	0.0456 (5)	0.0431 (5)	−0.0023 (4)	−0.0030 (4)	0.0026 (4)
C2	0.0337 (5)	0.0427 (6)	0.0405 (6)	−0.0006 (4)	−0.0012 (5)	0.0067 (5)
C4	0.0327 (6)	0.0474 (7)	0.0409 (6)	−0.0038 (5)	−0.0056 (5)	0.0060 (5)
C5	0.0299 (5)	0.0512 (7)	0.0473 (6)	−0.0002 (5)	−0.0042 (5)	0.0005 (6)
C6	0.0363 (6)	0.0466 (7)	0.0370 (6)	−0.0005 (5)	−0.0020 (5)	0.0043 (5)
C7	0.0480 (7)	0.0532 (7)	0.0609 (9)	−0.0052 (6)	−0.0075 (7)	−0.0063 (7)
C8	0.0427 (7)	0.0624 (9)	0.0638 (9)	0.0085 (6)	−0.0062 (7)	−0.0131 (8)
S1	0.0333 (1)	0.0475 (2)	0.0477 (2)	−0.0056 (1)	−0.0002 (1)	−0.0003 (2)
O3	0.0512 (5)	0.0429 (5)	0.0770 (8)	−0.0034 (4)	−0.0008 (5)	0.0019 (5)
O4	0.0348 (5)	0.0570 (7)	0.1024 (11)	−0.0026 (4)	−0.0073 (6)	−0.0052 (6)
O5	0.0665 (8)	0.1106 (12)	0.0446 (6)	−0.0298 (7)	0.0061 (6)	0.0007 (6)
C9	0.0424 (6)	0.0414 (6)	0.0427 (6)	−0.0060 (5)	−0.0025 (5)	−0.0021 (5)
C10	0.0417 (7)	0.0613 (9)	0.0742 (11)	−0.0056 (6)	0.0011 (7)	0.0173 (9)
C11	0.0507 (9)	0.0794 (12)	0.0814 (12)	−0.0191 (8)	0.0087 (9)	0.0105 (10)
C12	0.0847 (13)	0.0603 (9)	0.0493 (8)	−0.0265 (8)	0.0092 (8)	0.0011 (8)
C13	0.0933 (14)	0.0511 (9)	0.0649 (10)	−0.0095 (8)	−0.0067 (10)	0.0150 (8)
C14	0.0591 (10)	0.0530 (8)	0.0682 (10)	0.0010 (7)	−0.0086 (8)	0.0096 (8)
C15	0.123 (2)	0.0774 (13)	0.0754 (13)	−0.0454 (14)	0.0247 (14)	0.0022 (11)

### Geometric parameters (Å, °)

**Table:**

S1—O4	1.4538 (12)	C7—H7B	0.9600
S1—O5	1.4513 (16)	C7—H7C	0.9600
S1—C9	1.7618 (14)	C8—H8B	0.9600
S1—O3	1.4498 (12)	C8—H8C	0.9600
O1—C6	1.3269 (17)	C8—H8A	0.9600
O1—C8	1.4466 (18)	C9—C10	1.384 (2)
O2—C7	1.4386 (19)	C9—C14	1.376 (2)
O2—C4	1.3310 (17)	C10—C11	1.384 (3)
N1—C2	1.3560 (17)	C11—C12	1.385 (3)
N1—C6	1.3579 (16)	C12—C13	1.358 (3)
N2—C2	1.3210 (17)	C12—C15	1.517 (3)
N3—C4	1.3264 (17)	C13—C14	1.401 (3)
N3—C2	1.3438 (18)	C10—H10	0.9300
N1—H1	0.8600	C11—H11	0.9300
N2—H2B	0.8600	C13—H13	0.9300
N2—H2A	0.8600	C14—H14	0.9300
C4—C5	1.399 (2)	C15—H15A	0.9600
C5—C6	1.3638 (18)	C15—H15B	0.9600
C5—H5	0.9300	C15—H15C	0.9600
C7—H7A	0.9600		
			
S1···H2B	3.0600	C10···H7A^iv^	2.8700
S1···H2A^i^	2.9600	C10···H7B^i^	3.0900
S1···H1	2.8900	C11···H7A^iv^	2.9500
O1···C2^ii^	3.2870 (17)	C11···H15B^ix^	2.9600
O2···O3^iii^	3.1944 (17)	C15···H8C^x^	2.8300
O3···C5^iv^	3.3642 (18)	H1···O4	1.9000
O3···N2	2.9398 (18)	H1···S1	2.8900
O3···N2^i^	3.0233 (19)	H1···H2B	2.2700
O3···O2^iv^	3.1944 (17)	H2A···O5^vii^	2.7400
O4···N1	2.7441 (16)	H2A···O3^vii^	2.2000
O5···C5^ii^	3.356 (2)	H2A···S1^vii^	2.9600
O5···C8^ii^	3.248 (2)	H2B···O2^iv^	2.9100
O1···H15A^v^	2.8100	H2B···O3	2.1200
O2···H2B^iii^	2.9100	H2B···H1	2.2700
O3···H10	2.8700	H2B···S1	3.0600
O3···H2B	2.1200	H2B···H8A^viii^	2.5700
O3···H2A^i^	2.2000	H5···C8	2.6100
O4···H14	2.5600	H5···H8A	2.4000
O4···H1	1.9000	H5···H8B	2.4100
O5···H5^ii^	2.6700	H5···O5^viii^	2.6700
O5···H8B^ii^	2.3700	H7A···N3	2.7100
O5···H2A^i^	2.7400	H7A···C11^iii^	2.9500
O5···H7C^vi^	2.8300	H7A···C10^iii^	2.8700
N1···O4	2.7441 (16)	H7A···H10^vii^	2.5300
N1···C4^ii^	3.3492 (18)	H7B···C10^vii^	3.0900
N2···O3^vii^	3.0233 (19)	H7B···N3	2.5600
N2···O3	2.9398 (18)	H7B···H10^vii^	2.4100
N3···C6^viii^	3.3281 (17)	H7B···N3^viii^	2.8500
N2···H8A^viii^	2.7100	H7B···C2^viii^	2.7500
N3···H7B	2.5600	H7C···H8A^xiii^	2.5400
N3···H7A	2.7100	H7C···O5^xiv^	2.8300
N3···H7B^ii^	2.8500	H7C···C8^xiii^	3.0800
C2···O1^viii^	3.2870 (17)	H8A···C5	2.7900
C2···C7^ii^	3.449 (2)	H8A···H5	2.4000
C2···C6^viii^	3.4445 (19)	H8A···H7C^xii^	2.5400
C4···N1^viii^	3.3492 (18)	H8A···N2^ii^	2.7100
C5···O5^viii^	3.356 (2)	H8A···H2B^ii^	2.5700
C5···O3^iii^	3.3642 (18)	H8B···H5	2.4100
C6···C2^ii^	3.4445 (19)	H8B···O5^viii^	2.3700
C6···N3^ii^	3.3281 (17)	H8B···C5	2.7900
C7···C2^viii^	3.449 (2)	H8C···H15B^v^	2.5600
C7···C10^vii^	3.577 (2)	H8C···C15^v^	2.8300
C8···C15^v^	3.559 (3)	H10···O3	2.8700
C8···O5^viii^	3.248 (2)	H10···C7^i^	2.9000
C10···C7^i^	3.577 (2)	H10···H7A^i^	2.5300
C11···C15^ix^	3.583 (3)	H10···H7B^i^	2.4100
C15···C8^x^	3.559 (3)	H11···H15B	2.5400
C15···C11^xi^	3.583 (3)	H11···C7^i^	3.0600
C2···H7B^ii^	2.7500	H13···H15A	2.3800
C5···H8A	2.7900	H14···O4	2.5600
C5···H8B	2.7900	H15A···H13	2.3800
C7···H11^vii^	3.0600	H15A···O1^x^	2.8100
C7···H10^vii^	2.9000	H15B···H11	2.5400
C8···H7C^xii^	3.0800	H15B···H8C^x^	2.5600
C8···H5	2.6100	H15B···C11^xi^	2.9600
			
O5—S1—C9	105.93 (8)	H7B—C7—H7C	109.00
O3—S1—C9	107.10 (7)	H8B—C8—H8C	109.00
O3—S1—O4	111.62 (8)	O1—C8—H8A	109.00
O3—S1—O5	111.89 (9)	O1—C8—H8B	109.00
O4—S1—O5	113.38 (9)	O1—C8—H8C	109.00
O4—S1—C9	106.40 (7)	H8A—C8—H8B	110.00
C6—O1—C8	117.69 (11)	H8A—C8—H8C	109.00
C4—O2—C7	118.70 (11)	S1—C9—C10	117.83 (11)
C2—N1—C6	120.64 (11)	S1—C9—C14	122.21 (12)
C2—N3—C4	116.52 (12)	C10—C9—C14	119.92 (14)
C6—N1—H1	120.00	C9—C10—C11	119.68 (16)
C2—N1—H1	120.00	C10—C11—C12	121.23 (18)
C2—N2—H2B	120.00	C11—C12—C13	118.21 (18)
C2—N2—H2A	120.00	C11—C12—C15	120.0 (2)
H2A—N2—H2B	120.00	C13—C12—C15	121.76 (18)
N2—C2—N3	119.53 (12)	C12—C13—C14	121.99 (18)
N1—C2—N2	118.54 (12)	C9—C14—C13	118.94 (17)
N1—C2—N3	121.92 (12)	C9—C10—H10	120.00
O2—C4—N3	119.47 (13)	C11—C10—H10	120.00
N3—C4—C5	125.08 (13)	C10—C11—H11	119.00
O2—C4—C5	115.45 (12)	C12—C11—H11	119.00
C4—C5—C6	115.83 (12)	C12—C13—H13	119.00
O1—C6—N1	112.06 (11)	C14—C13—H13	119.00
N1—C6—C5	120.00 (12)	C9—C14—H14	121.00
O1—C6—C5	127.94 (12)	C13—C14—H14	121.00
C4—C5—H5	122.00	C12—C15—H15A	110.00
C6—C5—H5	122.00	C12—C15—H15B	109.00
H7A—C7—H7B	109.00	C12—C15—H15C	110.00
H7A—C7—H7C	109.00	H15A—C15—H15B	109.00
O2—C7—H7A	109.00	H15A—C15—H15C	110.00
O2—C7—H7B	109.00	H15B—C15—H15C	109.00
O2—C7—H7C	109.00		
			
O4—S1—C9—C10	−175.52 (13)	C4—N3—C2—N2	−177.98 (13)
O3—S1—C9—C10	−56.03 (14)	C2—N3—C4—O2	178.80 (12)
O3—S1—C9—C14	126.16 (13)	O2—C4—C5—C6	−179.77 (12)
O5—S1—C9—C14	−114.27 (14)	N3—C4—C5—C6	−0.5 (2)
O4—S1—C9—C14	6.66 (15)	C4—C5—C6—N1	0.8 (2)
O5—S1—C9—C10	63.54 (14)	C4—C5—C6—O1	−178.16 (13)
C8—O1—C6—N1	177.13 (12)	S1—C9—C10—C11	−177.39 (15)
C8—O1—C6—C5	−3.9 (2)	C14—C9—C10—C11	0.5 (3)
C7—O2—C4—N3	6.4 (2)	S1—C9—C14—C13	177.50 (14)
C7—O2—C4—C5	−174.33 (13)	C10—C9—C14—C13	−0.3 (2)
C2—N1—C6—C5	−0.07 (18)	C9—C10—C11—C12	0.6 (3)
C2—N1—C6—O1	179.01 (12)	C10—C11—C12—C13	−1.9 (3)
C6—N1—C2—N2	178.19 (13)	C10—C11—C12—C15	175.99 (19)
C6—N1—C2—N3	−1.0 (2)	C11—C12—C13—C14	2.1 (3)
C4—N3—C2—N1	1.2 (2)	C15—C12—C13—C14	−175.73 (19)
C2—N3—C4—C5	−0.4 (2)	C12—C13—C14—C9	−1.1 (3)

Symmetry codes: (i) −*x*+1, −*y*, *z*+1/2; (ii) −*x*+1/2, *y*, *z*+1/2; (iii) *x*−1/2, −*y*, *z*; (iv) *x*+1/2, −*y*, *z*; (v) −*x*+1, −*y*+1, *z*+1/2; (vi) *x*+1/2, −*y*, *z*+1; (vii) −*x*+1, −*y*, *z*−1/2; (viii) −*x*+1/2, *y*, *z*−1/2; (ix) −*x*+3/2, *y*, *z*+1/2; (x) −*x*+1, −*y*+1, *z*−1/2; (xi) −*x*+3/2, *y*, *z*−1/2; (xii) −*x*, −*y*, *z*+1/2; (xiii) −*x*, −*y*, *z*−1/2; (xiv) *x*−1/2, −*y*, *z*−1.

### Hydrogen-bond geometry (Å, °)

**Table:**

Cg is the centroid of the C9–C14 ring.

**Table:**

*D*—H···*A*	*D*—H	H···*A*	*D*···*A*	*D*—H···*A*
N1—H1···O4	0.86	1.90	2.7441 (16)	169
N2—H2A···O3^vii^	0.86	2.20	3.0233 (19)	161
N2—H2B···O3	0.86	2.12	2.9398 (18)	159
C8—H8B···O5^viii^	0.96	2.37	3.248 (2)	152
C7—H7A···Cg^iii^	0.96	2.96	3.7815 (18)	145

Symmetry codes: (vii) −*x*+1, −*y*, *z*−1/2; (viii) −*x*+1/2, *y*, *z*−1/2; (iii) *x*−1/2, −*y*, *z*.
